# Prediction of major liver-related events in the population using prognostic models

**DOI:** 10.1093/gastro/goaf028

**Published:** 2025-03-14

**Authors:** Fredrik Åberg, Ville Männistö

**Affiliations:** Transplantation and Liver Surgery Unit, Helsinki University Hospital and University of Helsinki, Helsinki, Finland; School of Medicine, Institute of Clinical Medicine, University of Eastern Finland, Kuopio, Finland; Department of Medicine, Kuopio University Hospital, Kuopio, Finland

**Keywords:** liver-related events, prediction, prognosis, cirrhosis, chronic liver disease, CLivD

## Abstract

Liver disease poses a significant global health burden, with steatotic liver disease related to metabolic dysfunction and/or alcohol use being the most prevalent type. Current risk stratification strategies emphasize detecting advanced fibrosis as a surrogate marker for liver-related events (LREs), such as hospitalization, liver cancer, or death. However, fibrosis alone does not adequately predict imminent outcomes, particularly in fast-progressing individuals without advanced fibrosis at evaluation. This underscores the need for models designed specifically to predict LREs, enabling timely interventions. The Chronic Liver Disease (CLivD) risk score, the dynamic aspartate aminotransferase-to-alanine aminotransferase ratio (dAAR), and the Cirrhosis Outcome Risk Estimator (CORE) were explicitly developed to predict LRE risk rather than detect fibrosis. Derived from general population cohorts, these models incorporate either standard liver enzymes (dAAR and CORE) or risk factors (CLivD), enabling broad application in primary care and population-based settings. They directly estimate the risk of future LREs, improving on traditional fibrosis-focused approaches. Conversely, widely used models like the Fibrosis-4 index and newer ones, such as the LiverRisk and LiverPRO scores, were initially developed to detect significant/advanced fibrosis or liver stiffness. While not designed for LRE prediction, they have later been analyzed for this purpose. Integrating fibrosis screening with LRE-focused models like CLivD, dAAR, and CORE can help healthcare systems adopt proactive, preventive care. This approach emphasizes identifying individuals at imminent risk of severe outcomes, potentially ensuring better resource allocation and personalized interventions.

## Introduction

Liver disease is a significant contributor to global morbidity and mortality, with steatotic liver disease (SLD) and its subtypes—metabolic dysfunction-associated steatotic liver disease (MASLD, previously known as NAFLD) and alcohol-related liver disease (ALD)—being the most common chronic liver diseases. Together, these subtypes affect up to 30%–40% of the global population [1, 2]. Despite the high prevalence of MASLD, alcohol remains the leading driver of severe clinical outcomes, creating a “burden paradox” where the most prevalent disease subtype contributes less to advanced outcomes compared to ALD [3]. Understanding this disparity is critical for improving liver disease management strategies.

Most individuals with significant liver disease remain undetected until they experience liver-related events (LREs), such as hospitalization, liver cancer, or death [4]. At this point, prognosis is often poor, underscoring the need for early detection and intervention [5]. The progression of liver disease typically follows a continuum, from fibrosis to cirrhosis, and ultimately to LREs. This progression is driven by the cumulative impact of multiple risk factors, including metabolic factors, alcohol use, and genetic predisposition, often acting together in complex and synergistic ways [6].

Current screening tests are designed to determine whether an individual has advanced fibrosis at the time of evaluation, but fibrosis is merely a surrogate marker for the outcomes that matter most—clinical LREs. While fibrosis is a key predictor of LREs, it does not always capture the full risk spectrum. Some individuals with advanced fibrosis may progress slowly over many years (“slow progressors”), while others with earlier fibrosis stages may experience rapid progression to severe outcomes (“fast progressors”) [7]. This variability highlights the limitations of relying on fibrosis alone to identify individuals at high risk of imminent LREs.

Recently, some prediction models have also been developed and validated to directly assess the risk of future LREs in the general population [8, 9]. These models offer the potential for tailored interventions and population-level risk stratification, complementing traditional fibrosis-based approaches. This narrative review discusses the difference between fibrosis testing and LRE prediction, summarizes the existing models for predicting LREs in the community, and evaluates their clinical applicability. We also discuss the potential of these prediction models to shift the paradigm toward proactive and preventive care.

## Current concepts of fibrosis screening as recommended by guidelines

Current guidelines for liver disease management advocate fibrosis screening in at-risk populations, such as individuals with metabolic risk factors or hazardous alcohol use, but not in unselected general populations [10, 11]. Among these, the Fibrosis-4 (FIB-4) index is widely recommended as a first-line test due to its low cost and accessibility [12]. FIB-4 uses age and routine laboratory data (aspartate aminotransferase [AST], alanine aminotransferase [ALT], and platelet count) to stratify fibrosis risk and identify individuals who may benefit from further evaluation [10, 11].

However, FIB-4 has notable limitations. It produces a high rate of false-positive results, particularly in individuals over 65 years, and is unreliable in younger populations under 35 years [12, 13]. Moreover, its accuracy diminishes in general population settings where advanced fibrosis prevalence is low, making it less effective as a universal screening tool [5]. For individuals with intermediate FIB-4 values, second-line tests, such as elastography or the enhanced liver fibrosis (ELF) test, are recommended.

Several newer fibrosis scores, such as LiverRisk, LiverPRO, steatosis-associated fibrosis estimator (SAFE), and metabolic dysfunction–associated fibrosis (MAF-5), aim to address these limitations by incorporating additional variables and design within larger populations [14–17]. However, most of these scores require further validation in diverse populations and in sequential testing strategies. Furthermore, the cost-effectiveness of these newer tools remains uncertain and may vary based on clinical context and country.

Despite the utility of fibrosis screening in identifying individuals with advanced disease, it might be limited in predicting the outcomes that matter most—clinical LREs. Fibrosis screening tends to favor slow progressors who have had years to accumulate advanced fibrosis while potentially missing fast progressors who may experience severe outcomes without advanced fibrosis at the time of evaluation. This highlights the need for complementary risk prediction models that focus on identifying individuals at imminent risk of LREs.

By integrating fibrosis screening with broader risk prediction models, clinicians can improve stratification strategies. For example, models like the Chronic Liver Disease (CLivD) risk score [8] or Cirrhosis Outcome Risk Estimator (CORE) [18], which directly predict LRE risk, could serve as initial screening tools. Individuals identified as high-risk could then undergo fibrosis testing to refine risk estimates and guide personalized management. Such an approach could potentially help optimize resource allocation, reduce unnecessary testing, and focus interventions on those most likely to benefit.

## Published models for prediction of LRE in the general population

**Figure 1.**
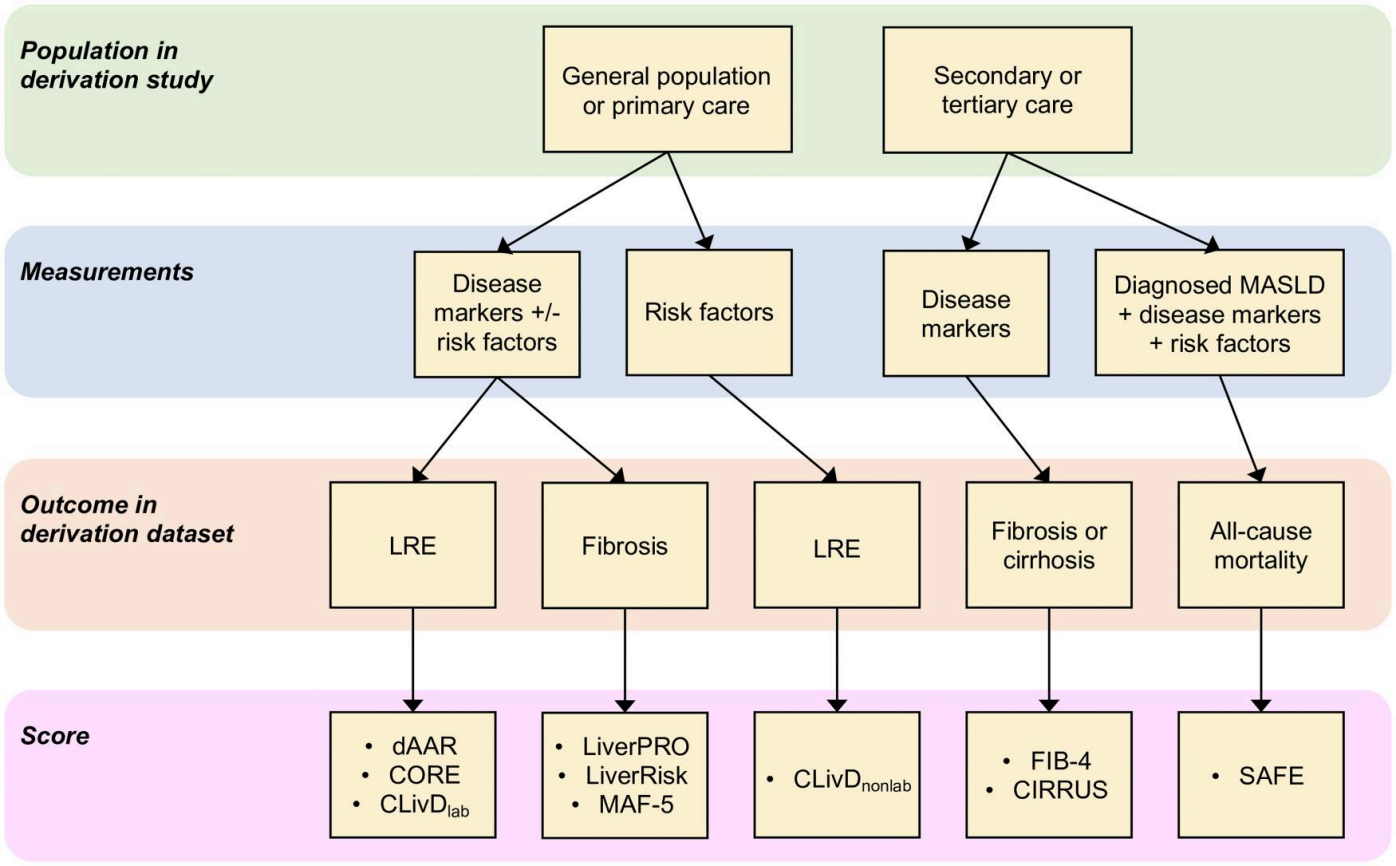
Existing models to predict the risk for liver-related events in the population categorized by the type of population used in the derivation of the score, measurements/components of the score (disease markers vs risk factors/disease drivers), and the primary outcome in derivation.

In this review, we focus on risk prediction scores that are based on variables widely available in primary care and that have been evaluated at the population level for predicting clinical LREs in independent cohorts. We prioritize models that are accessible and applicable in general practice settings, avoiding tools that require specialized laboratory measurements or liver elastography, as these may have limited scalability. Additionally, we include FIB-4 due to its widespread inclusion in clinical guidelines, despite its original design for fibrosis detection rather than LRE prediction. Key models, their design, and their performance in predicting LREs are summarized in Table 1 and Figure 1.

**Table 1.** Summary of main risk models designed or validated for prediction of liver-related events within general populations

Score	Derivation population characteristic	Outcome in derivation dataset	Variable	Availability	Discrimination performance in derivation sample	Independent validation for LRE in population-based cohort
**Scores derived in secondary or tertiary care datasets**						
FIB-4 [19]	International cohort of hepatitis C/HIV coinfected patients, *n* = 832	Liver fibrosis (Ishak stages 0–3 vs 4–6)	Age, ALT, AST, platelets	Open access	AUROC 0.77 for advanced fibrosis	Extensively validated; AUROCs 0.67–0.81 for LRE For LRE, 10-year AUROC: 0.70 [20]
CIRRUS [30]	Hospital cohort from the UK, *n* = 16,967	Cirrhosis or portal hypertension	Albumin, creatinine, bilirubin, MCV, Na, platelets, and total protein	Open access	AUROC 0.90 for cirrhosis/portal hypertension	For LRE: UHS primary and secondary care cohort (*n* = 394,253) 5-year AUROC: 0.90, CHIE primary care cohort (*n* = 183,045) 5-year AUROC: 0.84
SAFE [16]	MASLD hospital cohorts from USA, *n* = 676	Significant fibrosis (≥2 NASH CRN)	Age, body mass index, diabetes status, platelets, AST, ALT, and globulin (total serum protein minus albumin)	Open access	AUROC 0.79 for significant fibrosis. Performance better than NFS and FIB-4	C-statistics 0.73 for long-term mortality in NHANES III (*n* = 11,953) both in 5 years and in 22.4 years. Hong Kong data (*n* = 4,603) AUROCs 0.72–0.78 for 5–15 years who developed LREs [31], NHANES 1999–2016 (*n* = 46,357) C-statistic 0.76 for all-cause mortality [32]
**Scores derived in general population or primary care datasets**						
LiverRisk [14]	Multinational fibrosis screening cohorts from general population, *n* = 6,357	Liver stiffness (6, 10, and 15 kPa)	Age, sex, platelets, fasting glucose, cholesterol, AST, ALT, and GGT	Proprietary	AUROC 0.83 for TE 10 kPa	For liver-related mortality in UK Biobank: *n* = 416,200, 10-year AUROC 0.90; external validation: *n* = 17,259, 10-year AUROC 0.88 [28]
LiverPRO [15]	Danes with past or current excessive alcohol use, *n* = 462	Significant fibrosis (≥2)	Age, platelets, AST, GGT, ALP, total cholesterol, sodium, INR, bilirubin, and albumin	Proprietary	AUROC 0.86 for significant fibrosis	For LRE in UK Biobank (*n* = 470,795): C-statistics 0.74–0.78
MAF-5 [17]	NHANES 2017–2020 from USA, *n* = 9,023	Liver stiffness ≥8 kPa	Waist circumference, body mass index, diabetes, platelets, and AST	Open access	AUROC 0.81 for liver stiffness ≥ 8 kPa, 0.86 for liver stiffness ≥ 12 kPa. Performance better than LiverRisk, NFS and FIB-4	NHANES III (*n* = 10,944) with median follow-up of 22.8-year, MAF-5 associated with all-cause mortality (HRs 1.28–1.59)
dAAR [9]	Finnish general population, *n* = 18,067	LRE	Age, ALT, AST	Open access	Internally validated C-statistic 0.81 for LRE AUROC for cirrhosis 0.80–0.83 in NAFLD, 0.80 in HCV, 0.71 in ALD	Cohorts (*n* = 126,941) from Sweden (C-statistics 0.72) and Denmark (C-statistics 0.77–0.84) for LRE South Korean general population (*n* = 512,749) 12-year AUROC 0.81 for liver mortality [33]
ClivD [8]	Finnish general population, *n* = 25,760	LRE				
CLivD_lab_			Age, sex, alcohol use, waist-hip ratio, diabetes, smoking, GGT	Open access	Internally validated AUROC 0.84 for 15-year LRE	For LRE in general population cohorts: Danish Copenhagen Heart Study (*n* = 3,049, 15-year AUROC 0.78), China Kadoorie Biobank (*n* = 504,009 HBV-negative individuals, C-statistic 0.68) [35] In UK Biobank, 10-year AUROC 0.80 for LRE; AUROCs 0.86, 0.90 and 0.82 for liver-related death, alcohol-related cirrhosis and HCC, respectively [26].
CLivD_non-lab_			Age, sex, alcohol use, waist-hip ratio, diabetes, smoking	Open access	Internally validated AUROC 0.82 for 15-year LRE	For LRE in general population cohorts: Danish Copenhagen Heart Study (*n* = 3,049, 15-year AUROC 0.65), UK Whitehall II (*n* = 5,058, 15-year AUROC 0.78), China Kadoorie Biobank (*n* = 504,009 HBV-negative individuals, C-statistic 0.65) [35]. In UK Biobank, 10-year AUROC 0.72 for LRE; AUROCs 0.78, 0.85, and 0.75 for liver-related death, alcohol-related cirrhosis, and HCC, respectively [26]
CORE [18]	Swedish general population, *n* = 480,651	LRE	Age, sex, ALT, AST, GGT	Open access	AUROC 0.88 for 10-year LRE	Ongoing

ALT = alanine aminotransferase, ALP = alkaline phosphatase, AST = aspartate aminotransferase, AUROC = area under the receiver operating characteristic curve, CHIE = care and health information exchange, CIRRUS = CIRRhosis Using Standard Tests, FIB-4 = Fibrosis-4, GGT = gamma-glutamyl transferase, HBV = hepatitis B virus, HCC = hepatocellular carcinoma, INR = international normalized ratio, LRE = liver-related event, MASLD = metabolic dysfunction-associated steatotic liver disease, MCV = mean corpuscular volume, Na = natrium, NASH CRN = nonalcoholic Steatohepatitis Clinical Research Network, NFS = NAFLD fibrosis score, NHANES = National Health and Nutrition Examination Survey, TE = transient elastography, UHS = University Hospital of Southampton.

### Fibrosis-4

FIB-4 was originally developed as a tool to predict the presence of liver fibrosis in patients co-infected with HIV and hepatitis C [19]. This retrospective study included 832 patients and analyzed variables significantly associated with liver fibrosis (Ishak score 0–1, 2–3, or 4–6) using multivariate logistic regression. The area under the receiver operating characteristic curve (AUROC) for FIB-4 was 0.77 for distinguishing between Ishak stages 0–3 and 4–6. Using a cutoff of <1.45, the negative predictive value for excluding advanced fibrosis was 90%, with a sensitivity of 70%. A higher cutoff of >3.25 yielded a positive predictive value of 65% and a specificity of 97% [19].

While initially developed to detect fibrosis in patients with established liver disease, FIB-4 has since been evaluated as a predictive tool for LREs in population-based cohorts. Its performance in predicting future LREs, such as cirrhosis and related complications, was assessed in the Swedish Apolipoprotein Mortality Risk (AMORIS) cohort, which included 126,941 individuals from the general population [20]. FIB-4 demonstrated a positive association with the risk of LRE. The C-statistics for FIB-4 in predicting LREs ranged from 0.67 to 0.74, with AUROC values between 0.63 and 0.74 over follow-up periods of 5–10 years. Notably, FIB-4 performed better in individuals with risk factors for MASLD. However, 58% of LREs occurred in individuals with low-risk FIB-4 scores at baseline, while only 19% occurred in those with high-risk scores, raising concerns about its sensitivity for identifying at-risk individuals [20]. In another analysis from the same cohort, individuals with consistently low FIB-4 scores over a 5-year period experienced significantly fewer LREs compared to those with persistently high FIB-4 scores. Nonetheless, 48% of all LREs occurred in the group with consistently low FIB-4 values, indicating poor sensitivity for detecting all high-risk individuals [21].

A study using the UK Clinical Practice Research Datalink GOLD database evaluated 44,481 individuals in a primary care setting [22]. Among patients with obesity and/or type 2 diabetes, the 10-year risk of LREs was stratified as 15% in the high-risk group (FIB-4 > 2.67), 3% in the indeterminate group (FIB-4 1.30–2.67), and 1% in the low-risk group (FIB-4 < 1.30). However, 53% of liver events occurred in the low-risk group, again highlighting limitations in FIB-4’s sensitivity [22].

In the TriNetX global federated research network, which analyzed 442,277 individuals and included 81,108 in the final analysis, FIB-4 was associated with various clinical outcomes, including all-cause mortality, progression to metabolic-associated steatohepatitis (MASH), cirrhosis, end-stage liver disease, hepatocellular carcinoma (HCC), and liver transplantation [23]. The highest adjusted hazard ratios (HRs) of FIB-4 ≥ 2.67 were observed for MASH (HR 5.8) and liver transplantation (HR 8.0).

The predictive value of FIB-4 for LREs was also supported by an international multicenter cohort study of 320 biopsy-verified MASLD patients [24]. The AUROC for predicting LREs was 0.81, comparable to the AST-to-platelet ratio index (APRI) but slightly lower than the NAFLD fibrosis score (NFS). In this study, 84% of LREs were observed in individuals with high-risk FIB-4 scores, underscoring the importance of proper target group definition in screening strategies.

A meta-analysis of 2,518 biopsy-verified MASLD patients from 25 studies reported AUROCs of 0.69–0.81 for composite outcomes including all-cause mortality, HCC, liver transplantation, or cirrhosis-related complications during follow-up periods of 3–10 years [25]. Importantly, only 56% of individuals who reached the primary endpoint had high-risk FIB-4 scores (>2.67), reflecting again its limitations in sensitivity.

One key observation is that FIB-4’s predictive performance improves when the target population is carefully defined. For instance, combining FIB-4 with risk stratification tools like the CLivD score enhances its ability to predict LREs [26]. However, real-world data indicate that only ∼40% of cirrhosis patients had FIB-4 scores exceeding the high-risk threshold (>2.67) at the time of diagnosis or in the years leading up to it [27]. Additionally, about one-quarter of cirrhosis patients had FIB-4 scores below the rule-out threshold (<1.3), meaning these cases would have been missed entirely under FIB-4-based diagnostic strategies [27].

### LiverRisk

The LiverRisk score was developed in an international prospective fibrosis screening cohort comprising 6,357 individuals from the general population across six countries [14]. Fibrosis evaluation in this cohort was performed using transient elastography. The LiverRisk score is calculated based on age, sex, platelets, fasting glucose, cholesterol, AST, ALT, and gamma-glutamyltransferase (GGT). The development cohort included individuals from Europe and Hong Kong, and the score was subsequently validated in two external cohorts: the Rotterdam Study and the LiverScreen Study [14]. LiverRisk outperformed FIB-4 in predicting liver stiffness ≥ 10 kPa, with AUROCs of 0.83 versus 0.68, respectively.

The LiverRisk score has also been associated with long-term outcomes. In the UK Biobank data, it was linked to 10-year liver-related mortality, achieving an AUROC of 0.90, which was superior to FIB-4’s AUROC of 0.84 [14].

Furthermore, the score has been independently validated for outcome prediction in the UK Biobank cohort. Here, the score predicted liver-related mortality with AUROCs of 0.95, 0.88, and 0.87 after 5, 10, and 15 years, respectively. However, in this study, also the accuracy of FIB-4 was surprisingly good with AUROCs of 0.92, 0.85, and 0.82, respectively [28].

More recently, the LiverRisk score was tested in tertiary care cohorts comprising 5,897 and 1,558 patients, respectively [29]. In these settings, the score failed to accurately predict liver stiffness measurements or improve the identification of compensated advanced chronic liver disease. Additionally, its ability to predict hepatic decompensation within 1–5 years was inferior to that of FIB-4 [29].

Unfortunately, the formula for the LiverRisk score is not publicly available, limiting transparency and independent validations. However, an internet-based calculator has been provided for its application.

### LiverPRO

A Danish-led group recently introduced the LiverPRO score, a novel first-line fibrosis test for SLD associated with alcohol use and/or metabolic dysfunction [15]. The LiverPRO score incorporates age and nine routine blood tests: AST, GGT, alkaline phosphatase, total cholesterol, sodium, international normalized ratio (INR), bilirubin, albumin, and platelets. Importantly, the score is adaptable, requiring only age and between 3 and 9 analytes if all measurements are not available, enhancing its usability in various clinical settings.

The score was developed using data from 462 Danish individuals with current or past excessive alcohol use to detect biopsy-confirmed significant or advanced liver fibrosis. In the development cohort, LiverPRO demonstrated good discriminatory ability for significant fibrosis, with an AUROC of 0.86. External validation was performed across five European cohorts with varying risk profiles and disease prevalence, using liver stiffness measurements and/or LREs as comparators. The LiverPRO score showed moderate to good performance for detecting liver stiffness ≥ 8 kPa, with AUROCs ranging from 0.69 to 0.80 [15].

LiverPRO performed better in ALD than in MASLD, probably because it was developed among individuals with significant current or past alcohol use. Its diagnostic performance was comparable to the LiverRisk score and slightly better than FIB-4. For predicting LREs, LiverPRO achieved a C-statistic of 0.78 in the development cohort, which is comparable to other scores, such as FIB-4, NFS, ELF, and LiverRisk [15].

In the UK Biobank cohort, LiverPRO achieved a C-statistic of 0.74 for predicting LREs, outperforming FIB-4 (0.67) and marginally surpassing LiverRisk (0.72) [15]. Furthermore, in the development cohort, LiverPRO demonstrated a C-statistic of 0.87 for predicting liver-related mortality at 2 years. The LiverPRO has a CE approvement, but as a commercial product, the formula is not published.

### Metabolic dysfunction–associated fibrosis

MAF-5 score was developed and externally validated to assess fibrosis in individuals with metabolic dysfunction [17]. The development of the score utilized data from the population-based National Health and Nutrition Examination Survey (NHANES) 2017–2020 sample, while its validation was conducted using the population-based Rotterdam study (*n* = 5,967) and two hospital cohorts (*n* = 562 and *n* = 514). In the development sample, the AUROC of MAF-5 for predicting liver stiffness ≥ 8 kPa was 0.81, and for liver stiffness ≥ 12 kPa, it was 0.86. In the validation cohorts, the respective AUROCs ranged from 0.73 to 0.78 for liver stiffness ≥ 8 kPa and 0.73 to 0.85 for liver stiffness ≥ 12 kPa [17].

The prognostic utility of MAF-5 for all-cause mortality was evaluated using the NHANES III cohort, which included 10,944 individuals. Based on MAF-5 risk categories, 74% of participants were classified as low risk, 11% as intermediate risk, and 15% as high risk. Over a median follow-up of 22.8 years, 3,600 deaths were recorded. The MAF-5 score was significantly associated with all-cause mortality, with individuals in the intermediate-risk and high-risk categories demonstrating a higher mortality risk compared to the low-risk group (HRs 1.28–1.59), but no AUROCs were reported [17].

### CIRRhosis Using Standard Tests

The CIRRhosis Using Standard Tests (CIRRUS) model was developed using data from 16,967 individuals who underwent gastrointestinal endoscopy at the University of Southampton between years 2005 and 2016 [30]. The model aims to predict advanced liver disease and incorporates the following components: albumin, creatinine, bilirubin, mean corpuscular volume, sodium, platelets, and total protein. In the development cohort, CIRRUS demonstrated strong discriminatory performance for detecting cirrhosis, defined by either histology, liver stiffness measurement >15 kPa, serum fibrosis markers within the cirrhotic range, or portal hypertension, achieving an AUROC of 0.90 [30].

The CIRRUS model was validated in two large cohorts: one comprising primary and secondary care patients (*n* = 394,253) and another focusing solely on primary care patients (*n* = 183,045). For predicting severe LREs during a 5-year follow-up, CIRRUS achieved AUROCs ranging from 0.84 to 0.90 in the combined cohort and 0.83 to 0.88 in the primary care cohort, depending on whether the model was applied as a continuous or categorical variable [30].

Among patients with known risk factors for liver disease, such as alcohol use, diabetes, and viral hepatitis, CIRRUS predicted the occurrence of a first severe LRE with a sensitivity of 59%–72%, specificity of 87%–93%, positive predictive value of 18%–26%, and negative predictive value of 98%–99%. Across subsets of validation cohorts, CIRRUS demonstrated predictive performance for LREs that was comparable to that of the APRI and FIB-4 [30].

### Steatosis-associated fibrosis estimator

SAFE score was developed with the primary purpose of predicting the presence of significant liver fibrosis in individuals with MASLD [16]. The development cohort included 676 patients with biopsy-confirmed MASLD from the NASH Clinical Research Network. Validation was performed using the Farnesoid X nuclear receptor-ligand obeticholic acid for non-cirrhotic, non-alcoholic steatohepatitis trial cohort and an additional cohort undergoing magnetic resonance elastography [16]. The SAFE score formula includes age, body mass index, diabetes, platelets, AST, ALT, and globulins (total protein minus albumin). In the development cohort, SAFE demonstrated an AUROC of 0.79 for distinguishing fibrosis stage 0–1 from stage ≥2. Using a rule-out cut-off, sensitivity was 91% and the NPV was 84%.

The SAFE score’s ability to predict all-cause mortality was assessed in the NHANES III cohort (*n* = 11,953) [16]. SAFE achieved a C-statistic of 0.73 for mortality prediction within the 5 years, comparable to FIB-4 and NFS. Participants were categorized as low risk (SAFE < 0), intermediate risk (0 < SAFE < 100), and high risk (SAFE ≥ 100). Long-term follow-up revealed no significant differences in survival between those with SAFE < 0 and those without steatosis over 25 years. However, individuals with SAFE > 100 had significantly reduced survival (HR 1.53, C-statistics 0.73) for both 5-year and 22-year mortality outcomes [16].

Independent validation of the SAFE score was conducted among 4,603 MASLD patients in tertiary hospitals in Hong Kong [31]. For predicting LREs, SAFE achieved AUROCs of 0.72–0.78 over follow-up periods of 5–15 years. Using a high-risk cut-off of 100, the SAFE score accurately identified 85% of patients who developed LREs, compared to 41% for FIB-4 and 27% for APRI.

The SAFE score has also been associated with all-cause mortality in NHANES data spanning 1999–2016, where it was tested across different liver disease etiologies [32]. In this larger cohort (*n* = 46,357), SAFE achieved a C-statistic of 0.76 for predicting all-cause mortality, slightly outperforming FIB-4 and NFS (both at 0.74).

### Dynamic aspartate aminotransferase-to-alanine aminotransferase ratio

The dynamic AST-ALT ratio (dAAR) score was developed based on the hypothesis that the association between the AST-ALT ratio (de Ritis ratio) and liver fibrosis depends on the absolute levels of these transaminases [9]. The score was created using data from a Finnish population-based health examination survey (*n* = 18,067) linked to healthcare registries for LREs. External validation was conducted using the Swedish AMORIS cohort (*n* = 126,941). Additionally, the score was validated for predicting LREs, fibrosis, and cirrhosis in biopsy-confirmed cohorts from Sweden, Denmark, and the USA [9].

In the development cohort, dAAR demonstrated strong predictive performance for LREs, with an optimism-corrected C-statistic of 0.81. It also identified individuals with an absolute 10-year risk of LREs exceeding 10%. In the validation cohorts, the dAAR score exhibited a C-statistic of 0.72 in the AMORIS cohort, 0.81 in a MASLD cohort, and 0.75 in an ALD cohort. For detecting cirrhosis, the score achieved AUROCs of 0.80–0.83 in MASLD patients, 0.80 in hepatitis C patients, and 0.71 in ALD patients [9].

The dAAR score has since been independently validated in a South Korean general population cohort (*n* = 512,749). This study reported an AUROC of 0.81 for predicting liver-related mortality, further supporting the score’s prognostic utility [33].

### Chronic liver disease score

The CLivD score was developed as a straightforward tool to predict LREs in the general population [8]. Its design aimed for broad applicability across healthcare settings, with the added benefit of allowing individuals to calculate their risk independently via online calculators. The CLivD score incorporates age, sex, alcohol use, diabetes, waist-hip ratio, and smoking. While the addition of GGT enhances predictive performance, the score functions effectively even without requiring any laboratory tests.

In the development cohort, the CLivD score predicted LREs with a 15-year AUROC of 0.84 when including GGT (CLivD_lab_) and 0.82 without GGT (CLivD_non-lab_) [8]. External validation in the Copenhagen Heart Study (*n* = 3,049) yielded 15-year AUROCs of 0.78 for CLivD_lab_ and 0.65 for CLivD_non-lab_. In the British Whitehall II cohort (*n* = 5,058), the 15-year AUROC for CLivD_non-lab_ was 0.78 [8].

Further validation studies have examined the score’s performance. In the NHANES III cohort, the CLivD_lab_ score achieved a C-statistic of 0.75 for predicting liver-related mortality in Hispanics and 0.73 in the overall population, while the CLivD_non-lab_ score achieved a C-statistic of 0.64 in the overall population [34]. Similarly, in the Chinese Kadoorie Biobank, among hepatitis B virus (HBV)-negative individuals, the C-statistics were 0.68 for CLivD_lab_ and 0.67 for CLivD_non-lab_ [35].

In the UK Biobank cohort, 10-year AUROCs for predicting incident liver cirrhosis and cirrhosis-related complications were 0.80 (CLivD_lab_) and 0.72 (CLivD_non-lab_), compared to 0.75 for FIB-4 [26]. For alcohol-related cirrhosis, the corresponding AUROCs were 0.90 (CLivD_lab_), 0.85 (CLivD_non-lab_), and 0.79 (FIB-4). For predicting incident hepatocellular carcinoma, the AUROCs were 0.82, 0.73, and 0.75, respectively. Similarly, for liver-related mortality, the CLivD_lab_ score achieved an AUROC of 0.85 compared to 0.76 for CLivD_non-lab_ and 0.79 for FIB-4 [26]. Changes over time in the CLivD score reflect a corresponding change in the LRE risk [36].

### Cirrhosis outcome risk estimator

The CORE is the most recent addition to risk prediction models, designed specifically to estimate the 10-year risk of LREs in the general population [18]. The CORE score was developed using the Swedish AMORIS population-based cohort, which included 480,651 individuals with a median follow-up of 27 years. The parameters used in the CORE score are age, sex, AST, ALT, and GGT.

In its development cohort, CORE demonstrated strong predictive accuracy for LREs, achieving a 10-year AUROC of 0.88, significantly outperforming FIB-4, which had an AUROC of 0.79. So far, the CORE score has only been published in abstract form.

### Summary of the models

The risk prediction models differ in their derivation and validation populations, the variables used (risk factors vs disease markers), and the outcomes employed to calculate coefficients (Figure 1). They also vary in the methods for establishing cutoffs. Sensitivities and specificities for predicting LREs in general population or primary care cohorts are often inconsistently reported or omitted. While all of these models have been validated in independent cohorts, differences in methods, contexts, populations, and outcomes limit structured comparisons. Table 2 summarizes the findings from key studies that performed head-to-head comparison of some of the models for prediction of LRE in the general population setting. A large, population-based cohort with extended follow-up and comprehensive baseline data on disease severity is necessary for a robust head-to-head comparison of all the models. Most studies are based on Western populations, underscoring the need for research in diverse ethnic groups and healthcare systems. For example, while FIB-4 has been widely studied in various contexts and ethnic groups, of the newer models discussed, only CLivD and dAAR scores were reported to have been validated in the Chinese Kadoorie Biobank according to a recent systematic review [37]. Here, the CLivD score demonstrated strong predictive ability (C-statistic >0.7), though calibration adjustments might improve its accuracy [37]. Real-world effectiveness depends on defining appropriate target populations and integrating scores into clinical workflows.

**Table 2.** Key findings from independent general population cohorts (validation samples) reporting prediction of outcome events with head-to-head comparison of at least two models^a^

Author	Country	Participants	Outcome	Discrimination	PPV	NPV
Lindvig [15]	UK	470,795	LRE	5-year C-statistic FIB-4 0.73 (0.71–0.75) LiverPRO 0.77 (0.75–0.79) LiverRisk 0.76 (0.74–0.78)	n.r.	n.r.
Hydes *et al.* [30]	UK	6,105	LRE	5-year AUROC FIB-4 0.83 (0.80–0.86) CIRRUS 0.86 (0.83–0.88)	CIRRUS (crimson or red) + risk factors: 18%–26% CIRRUS (crimson or red) + no risk factors: 25%–4%	CIRRUS (crimson or red) + risk factors: 98%–99% CIRRUS (crimson or red) + no risk factors: 100%
Sripongpun *et al.* [16]	USA	11,953	All-cause mortality	C-statistic FIB-4 0.72 SAFE 0.73 (*P* < 0.01)	n.r.	n.r.
Åberg *et al.* [9]	Sweden	126,941	LRE	C-statistic FIB-4 0.71 dAAR 0.72	n.r.	n.r.
Åberg *et al.* [26]	UK	369,832	LRE	10-year AUROC FIB-4 0.75 (0.75–0.75) CLivD_lab_ 0.80 (0.80–0.80) CLivD_non-lab_ 0.72 (0.72–0.72) 10-year AUROC for alcohol-related cirrhosis outcomes FIB-4 0.79 (0.79–0.79) CLivD_lab_ 0.90 (0.90–0.90) CLivD_non-lab_ 0.85 (0.85–0.85)	FIB-4 1.30: 0.4% CLivD_lab_ low-high: 0.3% CLivD_lab_ interm-high: 2.6% CLivD_lab_ high: 5.4% CLivD_non-lab_ low-high: 0.3% CLivD_non-lab_ interm-high: 1.5% CLivD_non-lab_ high: 3.0%	FIB-4 1.30: 99.9% CLivD_lab_ low-high: 99.9% CLivD_lab_ interm-high: 99.9% CLivD_lab_ high: 99.8% CLivD_non-lab_ low-high: 99.9% CLivD_non-lab_ interm-high: 99.8% CLivD_non-lab_ high: 99.8%

a 95% confidence intervals are shown in the parentheses, where applicable.

AUROC = area under the receiver operating characteristic curve, CIRRUS = CIRRhosis Using Standard Tests, CLivD = Chronic Liver Disease, dAAR = dynamic aspartate aminotransferase-to-alanine aminotransferase ratio, FIB-4 = Fibrosis-4, LRE = liver-related event, NPV = negative predictive value, n.r. = not reported, PPV = positive predictive value, SAFE = steatosis-associated fibrosis estimator.

## Why we need prognostic models suitable for the population

Liver disease often progresses silently, making early identification of high-risk individuals challenging. Timely detection is critical, as lifestyle changes can slow disease progression and prevent LREs [38]. However, current screening approaches, which focus primarily on fibrosis detection, might be insufficient to meet this need. Fibrosis tests like FIB-4 have low sensitivity in general population settings [13]. Clear, evidence-based criteria for defining at-risk groups are needed to enhance the effectiveness of screening [39].

**Figure 2.**
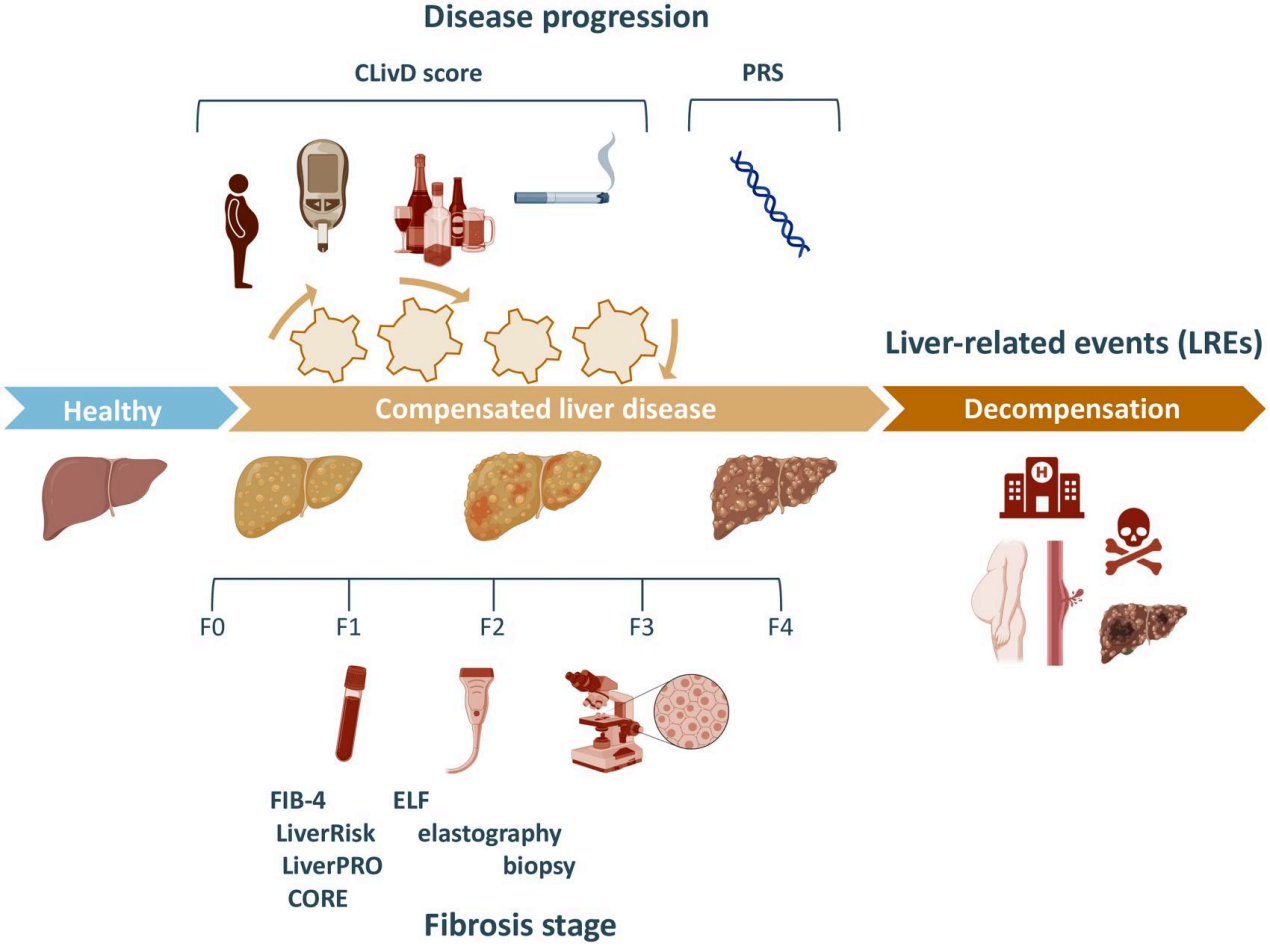
Conceptual framework of key drivers in chronic liver disease progression and fibrosis stage. Key disease drivers (risk factors) include alcohol use, smoking, obesity, diabetes, and genetic predisposition. The Chronic Liver Disease (CLivD) score quantifies the risk of liver-related events (LREs) based on the accumulation and severity of these drivers, while polygenic risk scores (PRSs) may quantify the impact of genetic risk variants. Fibrosis stage assessment provides a static snapshot of liver damage, indicating only the presence or absence of significant or advanced fibrosis at the time of evaluation. Figure created partly in https://BioRender.com.

**Figure 3.**
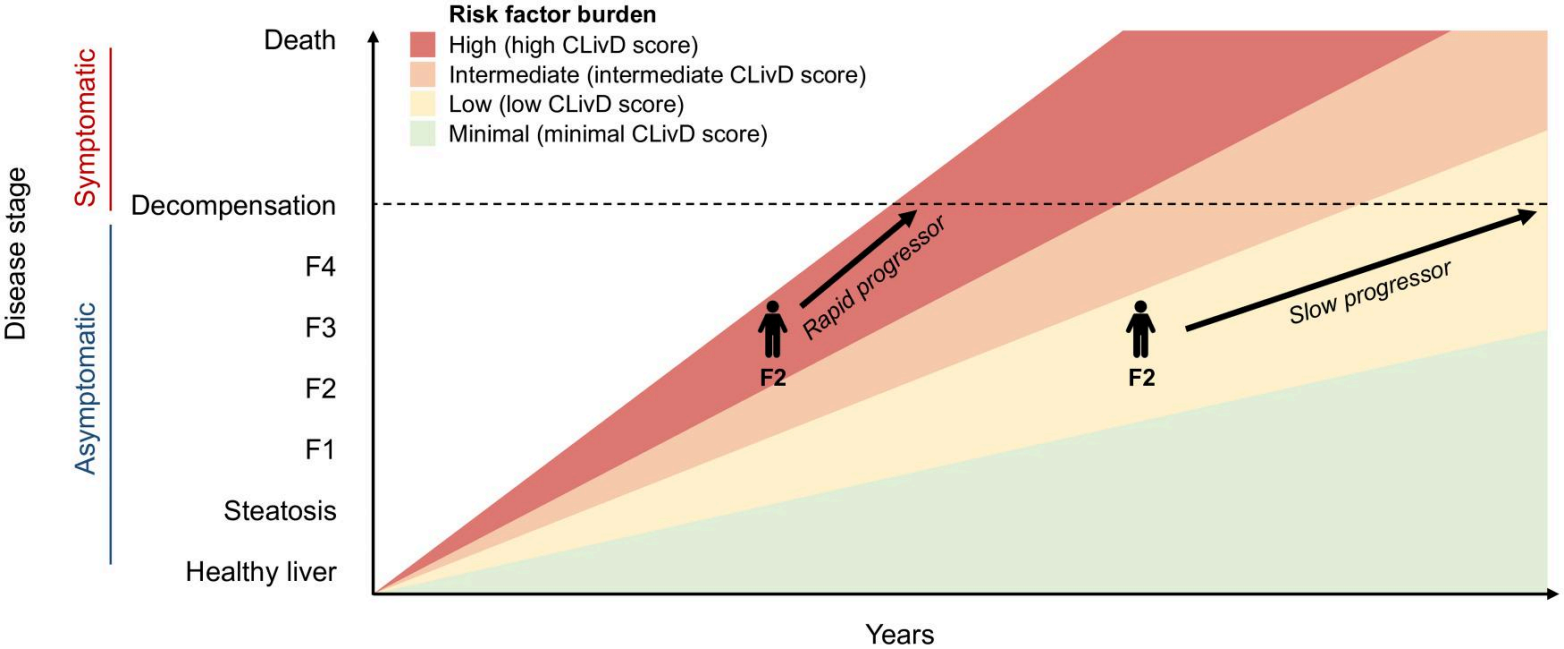
Conceptualization of the relationship between the Chronic Liver Disease (CLivD) score risk groups and time to cirrhosis decompensation and liver-related events. At any given fibrosis stage, the time to decompensation can vary considerably depending on the burden of risk factors, highlighting the importance of individualized risk assessment for such clinical liver-related events beyond fibrosis stage alone.

The pathophysiology of SLD is multifactorial, involving a complex interplay of alcohol consumption, metabolic risk factors, such as abdominal obesity and type 2 diabetes, and genetic predisposition [40] (Figure 2). Many individuals may have a combination of these factors, such as moderate alcohol use combined with metabolic risks and genetic risk variants, which cumulatively elevate their risk (Figure 3) [6, 40, 41]. Focusing on a single strong risk factor, such as severe obesity, is insufficient for identifying high-risk individuals, where the disease is driven by a combination of mild risk factors [6]. This complexity necessitates a comprehensive approach to risk stratification that accounts for the combined and synergistic effects of multiple contributing factors [6].

Models, like LiverRisk and LiverPRO, which lack publicly accessible formulae or rely on proprietary databases, pose challenges for independent validation, transparency, and widespread integration into routine clinical workflows. An ideal prognostic model for the population would be one that is low-cost, point-of-care, and widely accessible. Such a tool would facilitate large-scale risk stratification without requiring specialized equipment or invasive procedures. By enabling early identification of high-risk individuals, these models could guide timely interventions and allocate healthcare resources more effectively. Furthermore, using a widely available, open-access model would democratize risk assessment, enabling screening of large populations and integrating risk stratification into routine clinical practice across diverse socioeconomic settings. This is particularly important, as chronic liver disease is disproportionately prevalent in lower socioeconomic groups [42, 43], and accessible screening tools could help address this health disparity.

## Purpose: detection of subclinical disease versus prediction of future risk

There is an important conceptual difference between fibrosis-based prediction scores and risk factor-based scores in the context of liver disease. While fibrosis and cirrhosis are strong predictors of severe liver outcomes, they represent intermediate steps in the disease process rather than the ultimate endpoints. The real endpoints of interest are clinical LREs and patient-reported outcomes.

The majority of models currently recommended were originally designed to detect subclinical advanced disease, such as significant fibrosis (F2–4), which is often used as a surrogate marker for LRE risk. Although many of these models can also predict LREs, the majority of them were not specifically developed for this purpose. Consequently, detecting fibrosis may identify individuals with advanced liver disease, but it does not necessarily correlate with a high risk of imminent LREs. For example, the vast majority of those with stage F2–3 fibrosis will not progress to LREs within a clinically relevant timeframe [7]. A recent simulation model based mostly on biopsy studies estimated the 10-year risk of liver-related mortality in MASLD being 1.0% in F2 and 4.0% in F3 [44]. This can lead to unnecessary anxiety and distress for individuals labeled as having advanced liver disease without any clear benefit.

Additionally, currently recommended detection strategies seek to identify individuals who have already developed significant or advanced fibrosis [5, 40], thereby neglecting those, often younger individuals, who might progress to such stages in the near future. Liver disease progression varies widely, with some individuals experiencing rapid deterioration (fast progressors) while others remain stable for years (slow progressors) [7]. Fibrosis screening may, therefore, be more likely to identify slow progressing older individuals due to length-time bias associated with screening practices [5]. Predicting LREs directly, rather than concentrating on intermediate stages, such as fibrosis, may be a more effective approach because it is set to target the final, clinically relevant endpoints. The CLivD, CORE, and dAAR scores were developed with the purpose of predicting LREs directly.

Labeling an individual as having advanced fibrosis or cirrhosis, and therefore as having “disease,” may lead to significant adverse psychological effects, including stigma and anxiety, especially in cases of false positives [45–48]. In contrast, classifying individuals as “at risk” for LREs might generally be perceived as less definitive and emphasizes risk factor management rather than disease management [46, 49, 50]. Communicating risk status rather than a disease diagnosis can encourage patients to engage more proactively in lifestyle changes, reducing feelings of helplessness and promoting a sense of control over their health, thereby providing a less distressing pathway for patient care [46, 49, 50].

This philosophy is similar to cardiovascular disease (CVD) management strategies, such as those employed by the SCORE and Framingham risk scores [46, 49]. In CVD, population screening for subclinical coronary heart disease is not recommended [51]. Instead, the focus is on lifestyle modification and management of risk factors, i.e. primary prevention. For high-risk individuals, i.e. those with high calculated risk of future CVD events, selective use of diagnostic tools can help to better stratify risk and guide more aggressive preventive or therapeutic strategies [51].

Applying a similar framework to liver disease, focusing on the prediction of future LREs enables primary prevention and risk factor management ideally before the development of advanced fibrosis. This approach shifts the emphasis from detecting disease (fibrosis) to detecting risk (risk of future disease), facilitating proactive risk reduction strategies similar to those used in the CVD field [49, 50]. Importantly, it avoids labeling individuals as diseased, which can lead to stigma and anxiety, and instead classifies them as “at risk”—a designation that might be perceived as less distressing and promote a more constructive approach to risk reduction [49, 50]. Also, it could reduce the number of individuals being subject to invasive diagnostics, such as liver biopsy. Nonetheless, research specific to the liver disease context is still limited regarding the psychological aspects of labeling individuals as having liver disease or being at high risk.

Screening for advanced fibrosis could be reserved for those at particularly high risk of LREs, such as individuals with a calculated risk for LRE exceeding 5%–10% over 10 years. Such a risk level is considerably higher than among the average individual with MASLD [7]. An accumulation of risk factors combined with high fibrosis scores significantly increases the risk of LREs compared to either factor alone [26], potentially justifying more targeted and extensive screening for subclinical disease in such groups, while avoiding overburdening individuals with low risk through unnecessary screening and surveillance [46]. This nuanced approach may help optimize resource allocation and reduce unnecessary labeling and anxiety associated with a diagnosis of liver disease, while still focusing on those most likely to benefit from early intervention.

## Potential use of prediction models in clinical practice and advantages of risk-factor based scores

The use of tests for screening subclinical advanced liver fibrosis and referring patients to specialized care presents significant challenges. Current strategies risk overwhelming hepatology services due to the large number of patients requiring further investigation [52]. Moreover, most patients who develop LREs within the next 10 years fall into low-risk categories based on fibrosis scores like FIB-4 [20]. This paradox means that while specialized care becomes burdened with assessing disease stages, many future LREs are missed due to the limitations of current fibrosis tests, the risk stratification process and length-time bias of screening. While repeat testing may offer a slight improvement in accuracy [21], it also substantially increases the demand for testing resources.

Although some, but not all, previous studies have suggested that fibrosis screening in SLD can be cost-effective, these conclusions often rely on numerous assumptions, extrapolations, and expert opinions [53]. Even when considered cost-effective based on country-specific willingness-to-pay thresholds, screening still imposes substantial costs on society and healthcare systems, including opportunity costs [54].

It is essential that fibrosis screening be targeted toward individuals at high risk for LREs. Traditional risk factors to determine such “high risk,” such as harmful alcohol use and metabolic syndrome, when considered in isolation, lack sufficient sensitivity and specificity for this purpose [52]. The CLivD score probably presents a more effective tool for this purpose, as it quantifies LRE risk based on the cumulative number and severity of various risk factors within an individual [52].

In general, clinical practice guidelines aim to enhance the quality and efficiency of care, but they often do not account for the substantial clinician time required to implement all recommendations. For example, it has been estimated that fully adhering to all preventive care guidelines in the USA would require primary care physicians to work up to 27 hours a day [55]. Implementing extensive liver fibrosis screening with repeated testing over time would likely add to such unsustainable demands on healthcare systems, diverting time and resources from other important aspects of patient care.

The CLivD score offers a cost-free, accessible tool for large-scale risk stratification without requiring physician visits or contacts. It allows individuals to monitor their risk and engage in preventive lifestyle changes. The use of the CLivD score thus makes it possible to align risk stratification with behavior change.

Indeed, a previous study showed that changes in CLivD scores over time predicted LREs independent of baseline CLivD scores, highlighting the value of ongoing monitoring [36]. Although not all high-risk patients identified by the CLivD score will develop LREs, implementing lifestyle interventions, such as alcohol reduction, smoking cessation, and weight loss, offers broader health benefits beyond liver disease. This targeted approach has the potential to enhance the utility of risk stratification and minimize unnecessary interventions, ultimately improving overall health outcomes. Moreover, it could help integrate liver-oriented risk stratification and primary prevention into existing primary-care frameworks for the management of long-term conditions, such as metabolic disorders, type 2 diabetes, and alcohol use disorder. Making better use of such existing frameworks, considering liver health routinely alongside other chronic conditions, could help increase the interest and engagement of primary care physicians in liver-based risk stratification and promote a more holistic approach to management.

FIB-4 and many other tests designed to detect significant or advanced fibrosis do not have a linear association with LRE risk [56]. As a result, the rate of change of FIB-4 over time does not consistently predict LREs, limiting its usefulness as a monitoring tool [56, 57].

Moreover, misinterpretation of normal fibrosis test results, such as those from FIB-4 or liver stiffness measurements, can lead to a false sense of security. Individuals with normal fibrosis test results may continue harmful behaviors like alcohol consumption and a sedentary lifestyle, believing they are not at risk. This highlights the importance of using risk prediction models that focus on future LREs rather than current fibrosis stage alone.

By using the CLivD score as a first-tier risk stratification method, followed by fibrosis testing only for those at particularly high risk, we could potentially reduce lead-time and length-time biases in liver fibrosis screening. This targeted strategy ensures that screening efforts focus on those most likely to benefit from early detection and intervention, avoiding unnecessary testing in low-risk individuals and reducing overdiagnosis.

## Practical considerations for implementation

Integrating risk prediction models with automated laboratory systems and clinical decision support tools can facilitate adoption in routine care and reduce training needs [58]. In contrast, the CLivD score can be calculated by individuals themselves using web-based calculators, without requiring blood tests. It includes waist-hip ratio, which can be accurately self-measured using a measuring tape or smartphone technology, thus ensuring accessibility and ease of use without specialized training.

## Conclusions and future directions

There are notable limitations with risk stratification based on models originally purposed for detection of significant or advanced liver fibrosis. While widely accessible, FIB-4 is better at detecting cirrhosis than F2-F3 fibrosis and has suboptimal ability to differentiate LRE risk in many populations. Newer fibrosis models, like LiverRisk and LiverPRO, seem to perform better in detecting advanced fibrosis but the advantage for predicting LREs over FIB-4 is less clear. The CORE model, designed specifically to predict LREs rather than fibrosis, seems a promising laboratory-based tool suitable for automated risk stratification, but full publication and further validation are awaited. Many of these scores need to be validated in diverse ethnic populations.

The CLivD score is based on a fundamentally different approach, aiming to identify individuals at risk of future LREs in the general population by assessing the severity and accumulation of risk factors, rather than relying on laboratory tests that primarily detect advanced fibrosis. This approach, validated in diverse populations, has the potential to enable cost-effective, large-scale, digital-based risk stratification, where individuals can perform the risk prediction themselves.

Future large studies with comprehensive longitudinal data are crucial to enable head-to-head comparisons of existing predictive models across diverse populations and healthcare settings, ultimately identifying the best assessment strategy. Moreover, future research should focus on exploring novel markers of fibrosis stage, the potential integration of genetic data into risk prediction, and biomarkers that reflect disease progression beyond static fibrosis staging.

Finally, for any risk stratification or primary prevention strategy to be successful, there must be sufficient awareness of chronic liver disease among both the general population and healthcare professionals. Increased education and outreach should focus on prevention measures, ensuring that lifestyle modifications and early interventions are prioritized to manage chronic liver disease effectively before it progresses to more advanced stages.

## Authors’ contributions

Both authors contributed equally to drafting of the paper and providing critical comments to the final version.
